# Klaus Hentschel 2023: Lichtquanten. Die Geschichte des komplexen Konzepts und mentalen Modells von Photonen (erweiterte 2. Auflage): Heidelberg: Springer-Spektrum, brosch., 388 S., 42 Abb., 37,99 €, ISBN: 978-3-662-66932-7

**DOI:** 10.1007/s00048-023-00374-x

**Published:** 2024-02-12

**Authors:** Oliver Passon

**Affiliations:** Fakultät für Mathematik und Naturwissenschaften, Bergische Universität Wuppertal, Wuppertal, Deutschland

Der Wissenschaftshistoriker Klaus Hentschel hat 2017 eine viel beachtete Geschichte des Lichtquants vorgelegt, die bereits 2018 für die englische Übersetzung leicht überarbeitet wurde. Nun ist sie in einer zweiten und deutlich erweiterten deutschen Auflage erschienen. Hentschel wählt dabei den bemerkenswerten Zugang, eine kombinierte Begriffs- und Ideengeschichte zu schreiben, zu der philologische, kognitionspsychologische und wissenschaftshistorische Perspektiven beitragen. Er unterscheidet dabei verschiedene semantische Schichten des Konzepts, die in einem komplexen Prozess der Anreicherung (Akkretion) erst allmählich zur Ausformung des Begriffs führen. Zusätzlich führt Hentschel sogenannte mentale Modelle von Lichtquanten ein, die in der Forschungspraxis wirksam waren und sind.

All dies klingt furchtbar gelehrt (und ist es auch), aber einen wichtigen Punkt drückt diese geologische Metapher von Schichten und Akkretion ganz anschaulich aus: Alte Schichten werden nicht abgelöst, sondern verdichtet. Zudem können sie bisweilen wieder ans Tageslicht gelangen; sie behalten also bis auf den heutigen Tag Wirksamkeit. Auf diese Weise entsteht ein sehr differenziertes Bild der historischen Entwicklung mit ihren Brüchen und Umdeutungen von Newton bis zur aktuellen Quantenfeldtheorie. Dies ist umso begrüßenswerter, da populäre als auch Lehrbuchdarstellungen häufig eine direkte (und naive) Entwicklungslinie von Einsteins Lichtquanten-Hypothese aus dem Jahr 1905 bis zum aktuellen Photon ziehen. Wichtig ist zudem, wieviel Raum Hentschel der experimentellen Forschung einräumt – vor allem bei der Diskussion moderner Versuche seit 1945 (Kap. 8).

Die Publikation habe ich eingangs mit dem Attribut „viel beachtet“ versehen, da zahlreiche Rezensionen zur ersten Auflage erschienen sind – beispielsweise von Alexander Blum, John Heilbron oder Eugene Hecht. Je nach Perspektive lobten sie den methodischen Zugriff und die Neubelebung eines Dialogs zwischen Physikgeschichte und Physik (Heilbron und Blum als Historiker) oder bemängelten eher die technischen Ungenauigkeiten (Hecht als Physiker). Zahlreiche Besprechungen bemerkten jedoch, dass die modernen Entwicklungen zu kurz gekommen seien.

Klaus Hentschel nutzt nun die zweite Auflage, um diversen Verbesserungsvorschlägen Rechnung zu tragen. Das Vorwort beschreibt diesen charmanten Dialog zwischen Autor und seinen Leserinnen und Lesern genauer und listet die 15 Unterkapitel, die bei diesem Prozess neu entstanden sind. Es ist keine kleine Ironie, dass wir dadurch die Anlagerung zusätzlicher Bedeutungsschichten auch bei Hentschels Argumentation beobachten können.

Konzentrieren wir uns im Folgenden auf die Erweiterungen der Neuauflage. Im 5. Kapitel zur Rezeptionsgeschichte geht Hentschel nun zusätzlich auf die frühe Quantenfeldtheorie ein (5.5). Neu ist ebenfalls der Abschnitt 5.6 zu semiklassischen Theorien, die zahlreiche vorgebliche Photon-Effekte mit dem kontinuierlichen Strahlungsfeld und der Schrödingergleichung erklären können. Dass in diesem Zusammenhang Hentschel die Schrödingergleichung eine typische Wellengleichung und „zweiter Ordnung in Raum und Zeit“ (184) nennt, ist allerdings ein erstaunlicher Fehler, da sie natürlich erster Ordnung in der Zeit ist. Ebenfalls erwähnt Hentschel hier, dass die semiklassische Theorie „lokal-realistische Annahmen über Photonen“ (186) mache – obwohl die semiklassische Theorie ja gerade auf eine Quantisierung des Strahlungsfeldes verzichtet, also gar keine Photonen einführt.

Vor allem das siebte Kapitel zu alternativen Theorien der Begriffsentwicklung ist in der zweiten Auflage auf knapp 40 Seiten angewachsen. Eigentlich erstaunlich, dass Hentschel den dünnen vier Seiten der ersten Auflage überhaupt den Status eines eigenen Kapitels gegeben hatte. Das Resümee seiner Analyse verschiedener Modelle der Begriffsentwicklung besteht jedoch darin, seinen eigenen Zugriff über mentale Modelle als überlegen darzustellen.

Weitere größere Eingriffe in den Text hat das 9. Kapitel erlebt, das sich der Frage widmet, welches mentale Modell des Photons heute angemessen sei. Dieses Kapitel erschien mir bereits in der ersten Auflage sehr überzeugend, aber die zusätzliche Darstellung der Debatte um Wellen- versus Teilchen-Ontologie (9.1) und virtuelle Teilchen (9.3) enthält viele zusätzlich Hinweise auf die physikphilosophische Debatte, die in der ersten Auflage vermisst wurden. Erstaunlich finde ich jedoch, dass bei all diesen Eingriffen in den Text in der Liste der semantischen Schichten (Kap. 3) die zwölfte Schicht „Photonen als virtuelle Austauschteilchen der QED“ nicht auch dem Titel nach abgeändert wurde. Schließlich kennt die QED nicht bloß „virtuelle“ Photonen – um nur ein Problem dieser Bedeutungsebene zu nennen.

Bei aller Kritik an Details ist Hentschels Text sehr lesenswert, quellenreich und voller wertvoller Einsichten. Nur eine Antwort auf die Frage „Was ist ein Photon?“ sollte man nicht erwarten.

